# Losing my loss aversion: The effects of current and past environment on the relative sensitivity to losses and gains

**DOI:** 10.3758/s13423-020-01775-y

**Published:** 2020-07-27

**Authors:** Tim Rakow, Nga Yiu Cheung, Camilla Restelli

**Affiliations:** Institute of Psychiatry, Psychology and Neuroscience – Department of Psychology, King’s College London, London, UK

**Keywords:** Decision-by-sampling, Preference construction, Context effects, Stability of risk preference

## Abstract

It is often assumed that most people are loss averse, placing more weight on losses than commensurate gains; however, some research identifies variability in loss sensitivity that reflects features of the environment. We examined this plasticity in loss sensitivity by manipulating the size and distribution of possible outcomes in a set of mixed gambles, and assessing individual stability in loss sensitivity. In each of two sessions, participants made accept-reject decisions for 64 mixed-outcome gambles. Participants were randomly assigned to conditions defined by the relative range of losses and gains (wider range of losses vs. wider range of gains), and the currency-units at stake (‘pennies’ vs. ‘pounds’). Participants showed modest but non-trivial consistency in their sensitivity to losses; though loss sensitivity also varied substantially with our manipulations. When possible gains had greater range than possible losses, most participants were loss averse; however, when possible losses had the greater range, *reverse* loss aversion was the norm (i.e., more weight on gains than losses). This is consistent with decision-by-sampling theory, whereby an outcome’s rank within a consideration-set determines its value, but can also be explained by the gamble’s expected-value rank within the decision-set, or by adapting aspirations to the decision-environment. Loss aversion was also reduced in the second session of decisions when the stakes had been higher in the previous session. This illustrates the influence of prior context on current sensitivity to losses, and suggests a role for idiosyncratic experiences in the development of individual differences in loss sensitivity.

**Electronic supplementary material:**

The online version of this article (10.3758/s13423-020-01775-y) contains supplementary material, which is available to authorized users.

## Introduction

Loss aversion refers to weighting losses more than equivalent-sized gains. It can explain many decision phenomena including conservatism in long-term investing and over-valuing one’s assets (Benartzi & Thaler, 1995; Kahneman, Knetsch & Thaler, 1991). Kahneman (2011, p.300) describes loss aversion as “the most significant contribution of psychology to behavioural economics” and Rozin and Royzman (2001) regard it as one illustration of a general negativity bias whereby negative stimuli and events have greater potency than positive ones. Baumeister, Bratlavsky, Finkenauer and Vohs (2001) argue that numerous developmental, clinical and cognitive observations reveal this negativity bias, including: priority for negative stimuli in impression formation and attention tasks, faster learning with punishment than reward, and the speedy acquisition of conditioned aversions.

Loss aversion is often discussed as a feature of many people’s information processing. Rozin and Royzman (2001) deem it an *information-processing bias*; and Tom, Fox, Trepel and Poldrack (2007, p.515) suggested loss aversion “may reflect a fundamental feature of how potential outcomes are assessed by the primate brain”. When outlining that rejecting a symmetric 50–50 gamble with equal-sized losses and gains denotes loss aversion, Kahneman and Tversky (1979, p.279) asserted that: “most people find [such] bets … distinctly unattractive”. Other work also treats loss aversion as a property of the individual decision maker, but examines this as an individual difference. Thus, while it is often stated that people *typically* weight losses twice as much as gains (Tom et al.*,* 2007), cognitive models of individual decision makers find that this ratio (the loss aversion coefficient) varies between individuals and has moderate stability over time (*r* ≈ .5; Glöckner & Pachur, 2012). This suggests that sensitivity to losses is a noteworthy individual difference, which we explore in this paper by examining loss sensitivity (within-subjects) across different environments.

Recent work argues that loss aversion is something that occurs *in some environments*, rather than something seen *in (most) people* because when the environment changes so too can the apparent relative weighting for losses and gains (Gal & Rucker, 2018). Thus, loss aversion is often reduced or absent in: low-stakes decisions, when participants learn option-payoffs through repeated experience, or when symmetric 50–50 gambles are embedded among less attractive gambles (Ert & Erev, 2013; Yechiam & Hochman, 2013). Walasek and Stewart (2015) experimentally examined this plasticity in behavioural responses to losses. Using decision-by-sampling theory (Stewart, Chater & Brown, 2006), they hypothesised that the distribution of losses and gains in a series of mixed gambles affects sensitivity to losses because an outcome’s valuation relies on its rank within its consideration-set. For example, $32 ranks ‘low’ if other to-be-evaluated amounts are Uniform($24,$80) but ranks ‘high’ within Uniform($12,$40). Consequently, a 50–50 gamble for ±$32 should be less attractive when +$32 ranks low in the distribution of gains encountered in other gambles, and/or when -$32 ranks high (in magnitude) within the distribution of possible losses. Walasek and Stewart corroborated this hypothesis in four experiments: loss aversion reduced, disappeared and, occasionally, reversed as a function of the ranges of losses and of gains encountered over 64 accept/reject decisions for 50-50 gambles.^1^

To better understand how an individual’s underlying preferences and his/her environment influence sensitivity to losses, we replicated and extended Walasek and Stewart (2015). We re-examine their range manipulation, but also manipulate the stakes by a factor of 100 because previous research finds that raising the stakes increases the degree of loss aversion (Ert & Erev, 2013). If either manipulation substantially affects participants’ relative sensitivity to losses and gains (e.g., alters their propensity to accept a 50–50 gamble for equal-sized losses and gains), this would imply that loss sensitivity is context dependent and therefore that loss aversion may not be fundamental to how people process potential outcomes. Additionally, we ran a second session of decisions (within-subjects) to assess within-participant stability in loss sensitivity across different decision environments. This provides further data on the extent to which sensitivity to losses reflects an individual’s dispositions for information processing, or the features of his or her environment.

## Method

### Task and participants

One-hundred-and-nine adult volunteers completed the same online decision task on two different days, with different stimuli defining each session’s decision-set. Additionally, 45 participants completed only the first session. Each session required 64 accept-versus-reject decisions for a 50–50 gamble comprising one loss-amount and one gain-amount (presented simultaneously; upper/lower position randomised).

### Design and procedure

Each session had a 2-by-2 between-subjects design. One factor manipulated the relative range of losses and gains (range condition). Consistent with Walasek and Stewart (2015), gains or losses spanned 24–80 in eight-unit increments (high range) or 12–40 in four-unit increments (low range). We thereby created two range conditions: high-gain range with low-loss range (HGR-LLR), and low-gain range with high-loss range (LGR-HLR). The gambles in each range condition represented all possible pairings of the designated loss-amounts and gain-amounts. These two range conditions were symmetric in the sense that the median ratio for gains/losses was 2.0 (0.5) in the HGR-LLR (LGR-HLR) condition; and the HGR-LLR (LGR-HLR) condition had 86% of gambles with positive (negative) expected value (EV) and 9% with negative (positive) EV.

The other factor, amount condition, manipulated the currency-units: outcomes were ‘pennies’ (low-amount condition; e.g., ‘–16p’) or ‘pounds’ (high-amount condition; e.g., ‘–£16’). We randomly allocated each participant to a condition for Session 1 (*S1*), and (at least 1 day later) to one of the three remaining conditions for Session 2 (*S2*). On completing *S1*, participants received a 7-digit match-code to enter in *S2*. Most participants (54%) had one night between sessions with 26% having two to three nights between.^2^

### Data transparency

Project-completion deadlines dictated our data-collection stopping rule: we recruited participants between 5 December 2018 and 1 March 2019. We report all manipulations and dependent measures, and any data exclusions together with their reasons. The data are available at https://osf.io/384jd/.

### Data analysis

As per Walasek and Stewart (2015), we computed a loss-aversion coefficient for each participant to represent the *relative* impact of losses and gains on their decisions. Regressing accept-reject decision on gain-amount and loss-amount for each session’s 64 decisions generated this binary logistic regression model:

$$ {\mathrm{Log}}_{\mathrm{e}}\left[\frac{\mathrm{P}\left(\mathrm{accept}\right)}{1-\mathrm{P}\left(\mathrm{accept}\right)}\right]={\upbeta}_{\mathrm{bias}}+{\upbeta}_{\mathrm{loss}}\ \left(\mathrm{loss}\right)+{\upbeta}_{\mathrm{gain}}\ \left(\mathrm{gain}\right) $$

The regression coefficients β_loss_ and β_gain_ represent the sensitivity to losses and to gains. The loss aversion coefficient (LA_coefficient_) was computed as the ratio: β_loss_/β_gain_. LA_coefficient_ > 1 represents loss aversion; e.g., LA_coefficient_ = 2 indicates losses weighted twice-as-much as gains and therefore we expect gambles to be rejected unless the gain amount exceeds twice the loss amount. LA_coefficient_ < 1 represents *reverse* loss aversion (greater sensitivity to gains than losses) such that, for example, we expect gambles for equal-sized losses and gains to be accepted. Because LA_coefficient_ is asymmetric around 1 (loss neutrality), we use log_10_(LA_coefficient_) when analyses (e.g., ANOVA) work best for interval-like data. For such analyses, *positive* log(LA_coefficient_) denotes *loss aversion*, while *negative* log(LA_coefficient_) indicates *reverse* loss aversion.

We report standardised effect size measures and 95% confidence intervals (CIs) wherever possible. CIs for medians and correlation coefficients were obtained in *SPSS* via 10,000 bootstrap samples using the bias-corrected and accelerated method.

Applying the data-exclusion policy described by Walasek and Stewart (2015) to each session, we excluded 21/154 (14/109) participants for Session 1 (Session 2). These exclusions mitigate for participants’ inattention, task misunderstanding, or preferences that cannot be modelled, and were: (1) the 5% of individuals with the worst regression fit (highest minus-2-log-likelihood); (2) those with a negative regression coefficient (β_loss_ or β_gain_); or (3) making 64 invariant decisions.

*The* Online Supplementary Materials provide additional details of the methods.

## Results and discussion

### The effect of range and amount manipulations on loss sensitivity

Table 1 summarises LA_coefficient_ by condition and session, and suggests that the range manipulation substantially influenced loss sensitivity. Around three-quarters of participants were loss averse in the HGR-LLR condition; and every 95% confidence interval (CI) for the median LA_coefficient_ in this condition excludes values below 1. In contrast, most participants showed *reverse* loss aversion in the LGR-HLR condition; and, with one exception, the 95% CI for the median LA_coefficient_ excludes values above 1. Based on these distributions of the LA_coefficient_, we expect that most participants reject 50–50 gambles for equal losses and gains in the HGR-LLR condition, though most accept such gambles in the LGR-HLR condition. Table 2 confirms this expectation. In contrast, loss sensitivity differs little by amount condition.

**Table 1:** Median [95% confidence interval] (inter-quartile range) *{n*_*cell*_*}* for the loss aversion coefficient by condition

Amount condition	Range condition		Both range conditions combined
	HGR-LLR: High-gain range with low-loss range	LGR-HLR: Low-gain range with high-loss range	
Session 1 (*S1*)			
High	1.54 [1.12,1.61] (1.00–2.25) *{n = 31}*	0.94 [0.67,1.00] (0.64–1.07) *{n = 31}*	1.01 [0.96,1.19] (0.77–1.58) *{n = 62}*
Low	1.34 [1.08,1.61] (0.95–1.79) *{n = 36}*	0.77 [0.63,0.99] (0.56–1.25) *{n = 35}*	1.03 [0.86,1.30] (0.69–1.63) *{n = 71}*
Both amount conditions combined	1.36 [1.13,1.61] (1.00–1.84) *{n = 67}*	0.85 [0.67,1.00] (0.59–1.09) *{n = 66}*	1.01 [0.98,1.12] (0.75–1.61) *{n = 133}*
Session 2 (*S2*)			
High	1.48 [1.02, 1.86] (1.00–2.32) *{n = 29}*	0.93 [0.74,1.09] (0.68–1.12) *{n = 20}*	1.04 [0.99,1.38] (0.82–1.77) *{n = 49}*
Low	1.31 [1.03,1.87] (0.92–2.00) *{n = 25}*	0.75 [0.58,0.81] (0.50–1.11) *{n = 21}*	1.04 [0.80,1.22] (0.73–1.53) *{n = 46}*
Both amount conditions combined	1.41 [1.05,1.85] (1.00–2.00) *{n = 54}*	0.79 [0.74,1.00] (0.52–1.12) *{n = 41}*	1.04 [1.00,1.14] (0.75–1.58) *{n = 95}*

**Table 2:** Acceptance rate for gambles with equal-size loss- and gain-amounts, by condition and session

	Session 1 (*S1*)			Session 2 (*S2*)		
Amount condition	Range condition		Range cond. combined	Range condition		Range cond. combined
	HGR-LLR	LGR-HLR		HGR-LLR	LGR-HLR	
High	25.9%	54.4%	40.5%	28.1%	43.5%	34.5%
Low	34.1%	78.1%	55.0%	30.9%	60.5%	45.7%
Both amount conditions combined	30.3%	66.2%	48.1%	29.4%	52.7%	40.1%

Includes all participants by session (*N*s of 154 and 109) including those excluded from LA_coefficient_ analyses

**Fig. 1:**
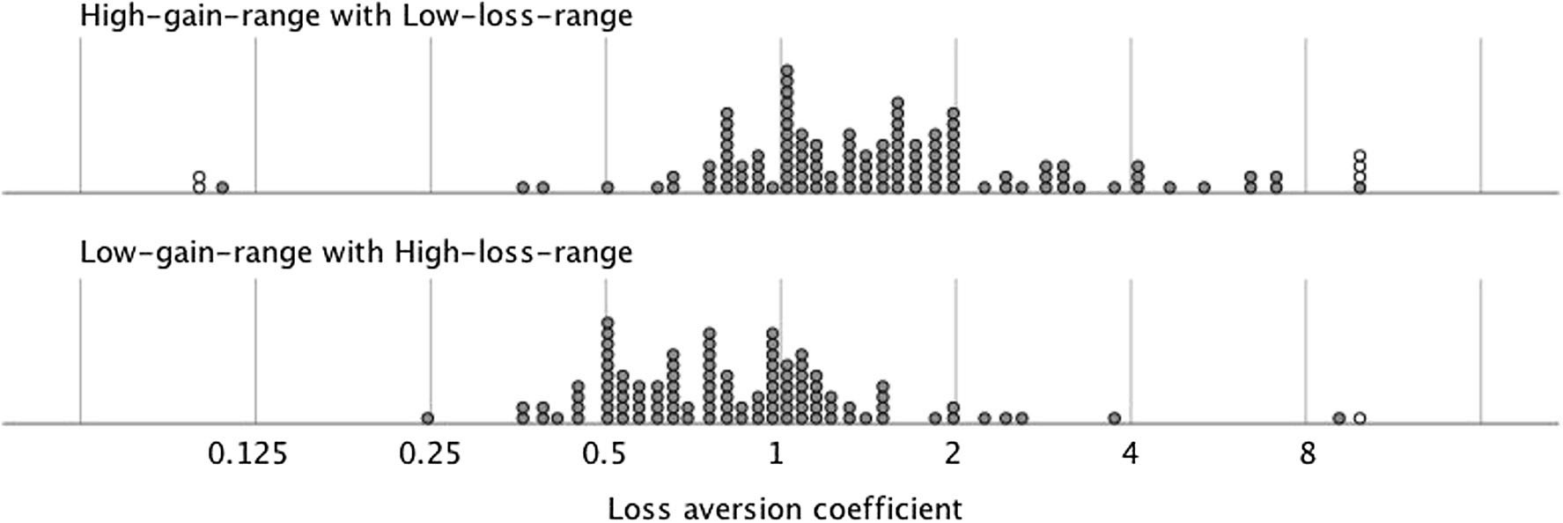
Loss version coefficient by range condition (HGR-LLR upper panel, LGR-HLR lower panel); data pooled from both sessions. *Note.* LA_coefficient_ is plotted on a logarithmic scale to preserve symmetry of the relative weight given to losses and gains, either side of equal weighting for losses and gains (LA_coefficient_ = 1). For example, LA_coefficient_ = 2 implies losses receive twice the weight of gains, while LA_coefficient_ = 0.5 implies losses receive half the weight of gains. Six outlying coefficients (shown as open icons) were truncated at 0.1 or 10 before being plotted

A two-way between-subjects ANOVA tested the effects of amount condition and range condition on log(LA_coefficient_) separately by session. There was a medium-sized and statistically significant main effect of range condition: *F*_*S1*_(1,129) = 19.81, *p* < .001, *η*_*p*_^2^ = .133 in Session 1 (*S1*); and *F*_*S2*_(1,91) = 8.86, *p* = .004, *η*_*p*_^2^ = .089 in Session 2 (*S2*); with higher log(LA_coefficient_) for HGR-LLR (*M*_*S1*_ = 0.16, *SD*_*S1*_ = 0.31; *M*_*S2*_ = 0.18, *SD*_*S2*_ = 0.43) than for LGR-HLR (*M*_*S1*_ = –0.06, *SD*_*S1*_ = 0.25; *M*_*S2*_ = –0.07, *SD*_*S2*_ = 0.34). Therefore, loss aversion was typical when the range of possible gains exceeded the range of possible losses; but *reverse* loss aversion was more common when the loss-range exceeded the gain-range. This effect is illustrated in Fig. 1, which plots LA_coefficient_ by range condition, with data pooled from both sessions. The effect of amount was small and non-significant in each session: *F*_*S1*_(1,129) = 0.57, *p* = .450, *η*_*p*_^2^ = .004; and *F*_*S2*_(1,91) = 1.93, *p* = .168, *η*_*p*_^2^ = .021; as was the two-way interaction, both *F* < 1, *p* > .57 and *η*_*p*_^2^ < .004.^3^ To further illustrate the effect of condition, Table 2 reports acceptance rates for the three 50–50 gambles with equal-size loss- and gain-amounts (±24, ±32 and ±40) that appeared in each condition. Only in decision-sets where the gain-range exceeded the loss-range (HGR-LLR condition) were such gambles consistently rejected (as would be expected if participants were loss averse). Under other circumstances – contrary to loss aversion – many participants accepted these gambles. The pattern of choice illustrated in Table 2 also runs counter to the predictions of rational choice theory because the propensity to accept a given gamble (e.g., a 50–50 gamble for ±32) varies between conditions. Thus, preferences change for a target choice when the range manipulation alters the *other* choices that the participant also makes.

Where possible, we matched LA measures across sessions.^4^ A within-subjects analysis (*N* = 88) of the effect of range condition corroborated our between-subjects analysis: loss aversion was reduced/reversed in the LGR-HLR condition when compared against the HGR-LGR condition, with the estimated effect size being slightly larger (*η*_*p*_^2^ = .150) than for the between-subjects analysis (see Online Supplementary Materials).

### Individual consistency in loss sensitivity

Next we used the matched LA measures to examine consistency in sensitivity to losses across sessions and conditions. Log(LA_coefficient_) correlated significantly across sessions, *r*(86) = .30, CI_95%_[.05,.51], *p* = .005, indicating some consistency in loss sensitivity across sessions – however, the Spearman correlation was not statistically significant, *ρ*(86) = .16, CI_95%_[–.07,.38], *p* = .130. These correlations might underestimate the consistency in loss sensitivity over time because there were several possible pairings of conditions that participants could be allocated to (see Online Supplementary Materials for correlational analyses by condition-pair). We therefore used partial correlation, controlling for the 12 possible combinations and orders of condition pair. Using the 11 dummy variables that this required, the partial correlation was positive, and statistically significant, *r*_*p*_(75) = .36, CI_95%_[.05,.61], *p* = .002.

### The effect of past experience on loss sensitivity

To explore whether past experience influenced subsequent decisions, we conducted a two-way between-subjects ANOVA with *S1* amount condition and *S1* range condition as factors, and log(LA_coefficient_) in *S2* as the dependent measure. There was a significant main effect of *S1* amount condition, *F*(1,84) = 6.97, *p* = .010, *η*_*p*_^2^ = .077, reflecting greater loss aversion in *S2* when participants had made decisions for low amounts (‘pennies’) in *S1* (*M*_*S2*_ = 0.18, *SD*_*S2*_ = 0.47) rather than for high amounts (‘£s’) in *S1* (*M*_*S2*_ = –0.04, *SD*_*S2*_ = 0.30). (the Online Supplementary Materials report this analysis for each condition.) Neither the main effect of *S1* range condition nor the two-way (range-by-amount) interaction were significant, both *F* < 1, *p* > .35, *η*_*p*_^2^ < .011.

### Three influences on loss sensitivity

Our findings point to three influences on an individual’s sensitivity to losses in a given set of decisions: (1) the distribution of losses and gains in that decision-set; (2) individual differences denoted by consistency across decision tasks; and (3) the size of losses and gains encountered in previous similar decisions. Table 3 summarises a regression model that tests these effects for independence, and estimates their relative contributions. Together, *S1* log(LA_coefficient_), *S1* amount condition, and *S2* range condition, account for 25% of the variability in *S2* log(LA_coefficient_), *F*(3,84) = 9.41, *p* < .001, R^2^ = .252. Each predictor is statistically significant.

**Table 3:** Multiple linear regression with log(LA_coefficient_) in Session 2 as the dependent variable

Predictor	Regression coefficients			Unique contribution beyond other predictors		
	Unstandardised *b* [95% CI]	Standardised *β*	*p*-value	R^2^ change	F_change_(1,84)	*p*-value
Constant	0.01 [–0.13,0.16]	--	.849	--	--	--
Log(LA_coefficient_) in Session 1	0.51 [0.23,0.78]	0.35	< .001	.122	13.73	< .001
Amount condition in Session 1	–0.19 [–0.35,–0.03]	–0.23	.020	.050	5.63	.020
Range condition in Session 2	0.24 [0.08,0.41]	0.29	.003	.081	9.05	.003

Amount condition: 0 = low, 1 = high

Range condition: 0 = low gain range with high loss range (LGR-HLR), 1 = high gain range with low loss range (HGR-LLR)

See the Online Supplementary Materials for a dominance analysis of how these predictors contribute to the regression model

Stability in loss sensitivity across sessions could reflect inertia (patterns in decision making established in *S1* continuing into *S2*) or stable tendencies within individuals. However, because this stability remains when controlling (via partial correlation) for allocation to condition-pairing, we assume it represents some stability in underlying preference rather than solely behavioural carry-over from *S1* condition.^5^

### Limitations

For 15/263 decision-sets (5.7%) we could not compute a LA_coefficient_ because the participant always made the same choice in that session. In most cases (13/15) all gambles were rejected, reflecting a relatively high degree of loss aversion. The majority of those always-reject cases (9/13) were in one condition: high amount (£s) and LGR-HLR. Thus, we have likely underestimated the degree of loss aversion in this condition. Accordingly, robustness checks using all participants (see Online Supplementary Materials) estimated a slightly smaller effect of range condition, and a slightly larger effect of amount condition than reported in Table 1, though the basic pattern was unchanged.

Due to sample-size limitations, there may be effects associated with particular *S1-S2* condition combinations that were not detected due to limited statistical power. Also, because each participant changed conditions across sessions, we missed collecting useful baseline data on test-retest reliability for loss sensitivity.

We were intrigued that *S1* amount condition predicted *S2* loss sensitivity. However, it was not a primary research aim to test this; rather, we manipulated amount to test its effect on loss sensitivity *within* a given set of decisions (not on subsequent decisions). Therefore, because this analysis was exploratory rather than confirmatory, this finding is provisional and needs further investigation.

### Conclusions

Reproducing Walasek and Stewart’s (2015) findings, the distribution of possible losses and gains in the decision-set altered loss sensitivity. When the range of gain-amounts exceeded the range of loss-amounts, we observed the ‘standard finding’: most participants exhibited loss aversion – seemingly, losses did *loom larger than gains* (Kahneman & Tversky, 1979). However, when the loss-range exceeded the gain-range, most participants showed reverse loss aversion. This reversal was not symmetric: loss aversion was more common in the HGR-LLR condition than was its reverse in the LGR-HLR condition (Table 1, Fig. 1) – even though the reversal of loss- and gain-distributions was symmetric across conditions. Other researchers find something similar: reversing the pattern of payoffs moderates ‘standard’ effects without necessarily creating an equal-sized effect in the opposite direction (Olivola & Sagara, 2009; Leuker, Pachur, Hertwig & Pleskac, 2018). For example, Walasek and Stewart (2019) found that reversing the direction of skew in asymmetric distributions of losses and gains attenuated loss aversion, but did not reverse it. Such attenuation-without-reversal can be understood in terms of the distributions of variables and their relationships outside the lab. For example, Stewart et al. (2006) illustrated how the ‘standard’ pattern of loss aversion is consistent with the observation that small losses (debits) are more common than small gains (credits) in personal transactions. Therefore, if participants judge value based on a mixture of experiences from inside and outside the lab, this will place bounds on how much patterns of preference (e.g., loss aversion) can be altered in the lab.

Our data add to the evidence that the distribution of possible losses and gains in a decision-set affects sensitivity to losses; but also provide a cautionary tale concerning how experimenters select study stimuli. For instance, Tom et al. (2007) examined the neural correlates of loss aversion using stimuli with ranges equivalent to those from our HGR-LLR condition, stating that: “We chose these ranges because previous studies indicate that people are, on average, roughly twice as sensitive to losses as to gains” (p.516). Their median LA_coefficient_ of 1.93 corroborate the previous research they cite – though our data illustrate that other stimuli would have likely generated a different degree of loss aversion. This does not mean that these stimuli were unsuitable for their study. It does, however, illustrate that when experimenters ‘tune’ stimuli to a behavioural phenomenon under investigation, they might also be tuning their participants’ behaviour.

In our experiment, losses and gains occurred with equal frequency, and the distributions of losses and gains were manipulated by altering their ranges. However, other work suggests that manipulating the *frequency* of losses and gains can affect loss aversion. For example, Yechiam and Rakow (2012) modelled individual-level data from six two-option repeated-choice tasks involving mixed outcomes, and included a model parameter reflecting the relative weighting for losses and gains. Mean values for this parameter implied loss aversion in tasks where gains were more frequent than losses, a lesser (minimal) degree of loss aversion when losses and gains were equally frequent, and clear reverse loss aversion when losses were more frequent than gains.^6^ Decision-by-sampling (Stewart et al., 2006) proposes this happens because the distribution of possible outcome amounts affects the subjective value of an individual outcome (e.g., a given loss-amount). However, this could also be explained at the level of the gamble and its EV (Ert & Erev, 2013). For example, 86% of gambles had strictly negative (positive) EVs in our LGR-HLR (HGR-LLR) condition. Thus, perhaps a 50–50 gamble for ±32 is frequently accepted in the LGR-HLR condition because it looks ‘good’ among so many negative-EV gambles, rather than because +32 looks ‘good’ and –32 looks ‘not-so-bad’ when compared against other possible outcomes in the set. Consistent with this, Ashby, Rakow and Yechiam (2017) observed that mixed gambles with negative EV were often chosen (over a zero sure-thing) in a three-option repeated-choice task when *both* non-zero options had negative EV.

Another possibility is that because positive-EV gambles are rare in the LGR-HLR condition, participants lowered their aspirations and accepted zero- or negative-EV gambles. Such adaptation looks like reverse loss aversion but could reflect demand characteristics: the participant feels they *should* accept *some* gambles and therefore takes the best on offer, even if these are unattractive options. Likewise, a ‘diligent’ participant in the HGR-LLR condition who rejects a reasonable number of the least attractive gambles may then reject some (otherwise-attractive) positive-EV gambles. Nonetheless, irrespective of exactly why it occurs, our data add to the evidence that the current decision environment can substantially influence people’s sensitivity to losses (Ert & Erev, 2013; Walasek & Stewart, 2015, 2019). However, our data also suggest that loss sensitivity is *not solely* a property of the current environment; two other potential influences were found.

First, loss sensitivity varied according to what choices participants encountered in a previous similar task. Seemingly, yesterday’s decisions for ‘pennies’ made today’s stakes seem ‘high’, and yesterday’s decisions for £s made today’s choices appear ‘low stakes’. Decision-by-sampling provides a theoretical framework for understanding this contrast effect. It assumes that potential outcomes are evaluated against outcome-values from the environment (e.g., the current task or choice set) as well as those sampled from memory. Consequently, decisions are not only influenced by possible outcome amounts encountered at the point of decision, but also by previously encountered amounts – especially those encountered recently. Decision-by-sampling also assumes that similarity influences which previously encountered values are sampled (Stewart et al., 2006, p.21). Although this aspect of the theory has not been extensively tested, it seems reasonable to assume that the similarity of the task performed in each session encouraged participants to sample memories from their previous session even though this was not particularly recent. However reasonable this assumption, it warrants further testing.

Additionally, loss sensitivity varied reliably between individuals. This stability was lower in our data than that estimated by Glöckner and Pachur (2012). Notably, while Glöckner and Pachur used independent decision-sets to model preferences, both sets had decisions with similar structure and payoffs. In contrast, payoffs differed considerably between our decision-sets, and therefore it is perhaps unsurprising that we found lower consistency within individuals. Nonetheless, while loss sensitivity was not highly stable across sessions, we regard the observed degree of consistency as meaningful. For example, the between-session correlation for loss sensitivity was modest (*r* = .30, *r*_p_ = .36), though similarly sized correlations are often found between items or indicators in multi-component measures of stable traits. We cannot be certain of the source of the consistency; however, the effects on loss sensitivity that we observed by manipulating current and recent decision environments highlight the possibility that stable components of risk preference may reflect an individual’s accumulated experience across a lifetime of idiosyncratic observations, experiences, decisions and incentives. Thus, whether the up-side or down-side of a decision seems ‘big’ or ‘small’, and therefore whether a risk is accepted, will depend upon an individual’s prior experiences (Stewart et al.*,* 2006). Olivola and Sagara (2009) demonstrated the plausibility of this: the country-specific distribution of single-event death-tolls predicted the degree of risk aversion for lives lost/saved in samples from those countries. Our data highlight the value of going beyond the aggregate analysis of such patterns to consider how an *individual’s* past experiences might shape their current risk preferences.

In sum, our experiment points to three potential influences on loss sensitivity: the current decision environment, recent decision environments, and individual risk preference (i.e., stable tendencies in loss sensitivity). Thus, sensitivity to losses for a given decision likely has a trait component (reflecting individual tendencies) and a state component (reflecting environmental features). Taken together, these findings imply that while some individuals may have a tendency to be loss averse, this tendency is sufficiently unstable, and the environmental influences are sufficiently powerful, that loss aversion *cannot* be regarded as a general feature of people’s decision making.

## Electronic supplementary material

ESM 1 (DOCX 182 kb)

## Footnotes

1 In addition to these empirical challenges to the loss aversion account, there are theoretical ones. For example, the loss attention account proposes that what looks like the effect *differential weighting* of losses and gains might be better understood as effects due to *increased attention* to decision tasks when losses are possible (Yechiam & Hochman, 2013).

2 Three participants completed both sessions on the same date: one completed *S1* just after midnight; two participants completed *S1* early morning and upon contacting us we likely assumed they had completed the day before.

3 It was not straightforward to use factorial ANOVA to examine effects (e.g., interactions) across sessions because our design was not fully factorial (i.e., no participant encountered the same condition twice).

4 Four participants typed *S2* match-codes that matched 6/7 digits of an allocated match-code; we judged this sufficient to match these data. One non-matched participant did not type their match-code in *S2*.

5 Supporting this interpretation, the regression coefficients in Table 3 barely altered when *S1* range condition was added as a fourth predictor.

6 Mean parameter values were: one to one-and-a-half standard errors (SEs) from loss/gain neutrality when gains were frequent; within three-quarters of a SE from loss/gain neutrality when losses and gains had equal frequency; and two-and-a-half to three SEs from loss/gain neutrality (in the opposite direction) when losses were frequent.
