# Supplementary material for: Losing my loss aversion: The effects of current and past environment on the relative sensitivity to losses and gains
Source: Psychon Bull Rev. 2020 Jul 27;27(6):1333–40. doi: 10.3758/s13423-020-01775-y (PMC7704442; doi:10.3758/s13423-020-01775-y)
Supplement: Supplementary file 1 — (DOCX 182 kb) [file 13423_2020_1775_MOESM1_ESM.docx]

**SUPPLEMENTARY MATERIALS**

**SUPPLEMENTARY MATERIALS 1: FULL METHOD WITH RATIONALE FOR DESIGN AND ANALYSIS**

**Task**

Participants completed the same decision task on two different days, with different stimuli defining their decision-set for each session. Participants read task details before providing consent, reading task instructions and commencing Session 1. Both sessions were conducted online, using *Qualtrics* survey software. Each session consisted of 64 monetary decisions (in random order), each being an accept-reject decision for a 50-50 gamble comprising a single loss-amount and single gain-amount. Each gamble was presented on one screen, with instructions above the loss/gain amounts: “Would you accept or reject the following 50/50 gamble?”. The loss/gain amounts were presented above/below each other (position randomised). Underneath this, participants selected a button: either “accept” (left) or “reject” (right).

**Design and procedure**

Each session had a 2-by-2 between-subjects design. One factor manipulated the relative range of losses and gains (range condition). Consistent with Walasek and Stewart (2015), gains or losses could range from 24 to 80 units in increments of 8 units (high range) or from 12 to 40 units in increments of 4 units (low range). Using this design feature, we created two range conditions with contrasting ranges for losses versus gains: a high gain-range with low loss-range condition (HGR-LLR), and a low gain-range with high loss-range condition (LGR-HLR). The 64 gambles in each condition represented all possible pairings of loss-amounts and gain-amounts for that condition. The other factor, amount condition, manipulated the currency-units for the gambles: outcomes were labelled as ‘pennies’ (low amount condition; e.g., “–16p”) or ‘pounds’ (high amount condition; e.g., “–£16”). Combining both factors factorially created four conditions. The survey software randomly allocated each participant to a condition for Session 1, and to one of the three remaining conditions for Session 2; and also generated an individual 7-digit code that the participant was asked to write down (to allow anonymous data-matching across sessions). The participant then contacted a researcher, who emailed them their Session 2 survey link the next day (ensuring a delay between sessions). In Session 2, participants typed their 7-digit match-code, were reminded of the task instructions, then made their 64 accept-reject decisions.

**Participants**

Adult participants (*N* = 154) were recruited from two (UK) university volunteer research panels (including staff and students) and via personal contact. Year 1 psychology undergraduates (*n* = 33) could receive credit for research participation, and the remaining participants were offered UK£4 for participating. One-hundred-and-nine participants completed both sessions.

**Data transparency**

Our stopping rule for data collection was dictated by external time constraints for project completion: we therefore recruited as many participants as possible between 05 December 2018 and 01 March 2019 (rather than aiming for a pre-determined sample size). We report all manipulations and dependent measures, and any data exclusions together with the reasons for these exclusions. The data are available.

**Data analysis**

To assess whether condition affected loss aversion, we followed the steps described by Walasek and Stewart (2015) to compute a loss aversion coefficient for each participant (separately for each session). This coefficient estimates the relative impact of losses and gains on an individual’s decisions. Consequently, binary logistic regression was used with decision (accept vs. reject) as the dependent variable, and gain-amount and loss-amount as the two predictors (modelling the 64 decisions in a session). This regression model can be represented as:

$$\frac{P\left( accept \right)}{1-P(accept)}=e^{\beta_{bias}+\beta_{loss}(loss) + \beta_{gain}(gain)}$$

Or, equivalently:

Log_e_[$\frac{P(accept)}{1-P(accept)}]=$ β_bias_ + β_loss_ (loss) + β_gain_ (gain)

The regression coefficients, β_loss_ and β_gain_, represent the sensitivity to losses and to gains across the 64 decisions. The loss aversion coefficient (LA_coefficient_) was computed as the ratio of these regression coefficients: β_loss_/β_gain_. Loss aversion coefficients above 1 represent loss aversion (greater sensitivity to losses than gains); e.g., LA_coefficient_ = 2 indicates losses weighted twice-as-much as gains. Coefficients below 1 represent *reverse* loss aversion (greater sensitivity to gains than losses); e.g., LA_coefficient_ = 0.5 represents weighting losses half-as-much as gains. The further the coefficient is from 1, the greater the disparity in the weighting of losses and gains; however, the LA_coefficient_ is asymmetric about its neutral point of ‘1’: coefficients in the range (1,∞) represent loss aversion, while coefficients in the range (1,0) represent *reverse* loss aversion. Consequently, the LA_coefficient_ does *not* have interval-like measurement properties, and is therefore poorly summarised by means or variances. Taking logs for LA_coefficient_ can address this; for instance, for the example-pair of LA_coefficient_ values above: log_10_(2) = +0.301, while log_10_(0.5) = –0.301. The symmetry of these values, either side of zero, reflects the complementary ‘meaning’ of LA_coefficient_ values of 2 and 0.5 (i.e., one input carries twice the weight of the other). We therefore use log_10_(LA_coefficient_) rather than LA_coefficient_ when analyses (e.g., ANOVA, Pearson *r*) work best for interval-like data. For such analyses, *positive* log(LA_coefficient_) denotes *loss aversion*, while *negative* log(LA_coefficient_) indicates *reverse* loss aversion.

We report standardised measures of effect size and 95% confidence intervals (CIs) wherever possible. The CIs for medians and correlation coefficients were obtained in *SPSS* via 10,000 bootstrap samples using the bias-corrected and accelerated method.

To support robust conclusions from the loss aversion (LA) measures, we followed the data-exclusion policy described by Walasek and Stewart (2015), applying it to each session separately We therefore excluded LA measures for the 5% of individuals with the worst regression fit (highest minus-2-log-likelihood): 8/154 (6/109) excluded from Session 1 (Session 2). These participants were those deemed least likely to be attentive or deliberative. We also excluded LA measures for any participant making 64 invariant decisions in a session (i.e., always-accept or always-reject) because the regression model could not fit these decisions. We excluded any participant with a negative regression coefficient for gains, or for losses, because such regression coefficients imply responding counter to the meaning of the stimuli: *viz.* increasing propensity to accept with increasing size of loss, and/or increasing propensity to accept with decreasing size of gain. In total, there were 21 (14) exclusions from Session 1 (Session 2).

**SUPPLEMENTARY MATERIALS 2: ADDITIONAL DATA ANALYSES**

**Robustness check on loss aversion coefficients**

For those participants previously excluded because their LA_coefficient_ could not be computed due to making the same decision through a session, we imputed extreme values for their LA_coefficient_ (beyond the range for those coefficients for other participants): 100 for always-reject, and 0.01 for always accept. Table S1 reports the recalculated summary statistics based on these data imputations, which can be compared against Table 1a in the main text. The main difference is in the values for the upper quartiles, which is often somewhat higher than in the original analyses. This is to be expected because most of these previously excluded participants are assumed to have a very high degree of loss aversion. However, the changes (from the original analyses) to the medians and their 95% confidence intervals (CIs) are much smaller. For Session 1, a small difference in median LA_coefficient_ emerges (greater loss aversion with high amounts); however, there remains considerable overlap in 95% CIs and the inter-quartile ranges of these two conditions. As anticipated, this new analysis provides a smaller estimate than before for the effect of range. Nonetheless, the difference between range conditions remains clear (e.g., non-overlapping 95% CIs between HGR-LLR and LGR-HLR conditions, in either session).

*Table S1.* Median [95% confidence interval] (inter-quartile range) for the loss aversion coefficient by condition and session.

| SESSION 1 | | | |
| --- | --- | --- | --- |
| Amount condition | Range condition | | Both range conditions combined |
|  | High gain range with low loss range (HGR-LLR) | Low gain range with high loss range (LGR-HLR) |  |
| High | 1.55 [1.11,1.74]  (1.00 to 2.70)  {*n* = 32} | 0.99 [0.91,1.07]  (0.65 to 1.74)  {*n* = 38} | 1.09 [1.00,1.42]  (0.79 to 2.33)  {*n* = 70} |
| Low | 1.33 [1.08,1.60]  (0.93 to 1.77)  {*n* = 37} | 0.75 [0.60,1.00]  (0.54 to 1.21)  {*n* = 36} | 1.00 [0.73,1.25]  (0.67 to 1.61)  {*n* = 73} |
| Both amount conditions combined | 1.36 [1.13,1.60]  (0.97 to 1.92)  {*n* = 69} | 0.93 [0.74,1.00]  (0.60 to 1.36)  {*n* = 74} | 1.06 [1.00,1.19]  (0.76 to 1.73)  {*N* = 143} |
| SESSION 2 | | | |
| Amount condition | Range condition | | Both range conditions combined |
|  | High gain range with low loss range (HGR-LLR) | Low gain range with high loss range (LGR-HLR) |  |
| High | 1.51 [1.02, 1.94]  (1.00 to 2.69)  {*n* = 30} | 0.99 [0.75,1.11]  (0.72 to 1.16)  {*n* = 22} | 1.10 [1.00,1.49]  (0.86 to 1.96)  {*n* = 52} |
| Low | 1.31 [1.06,1.85]  (0.92 to 2.00)  {*n* = 25} | 0.75 [0.54,1.10]  (0.50 to 1.16)  {*n* = 23} | 1.08 [0.82,1.25]  (0.75 to 1.77)  {*n* = 48} |
| Both amount conditions combined | 1.44 [1.10,1.85]  (1.00 to 2.01)  {*n* = 55} | 0.83 [0.75,1.09]  (0.54 to 1.15)  {*n* = 45} | 1.10 [1.00,1.22]  (0.76 to 1.86)  {*N* = 100} |

**Within-subjects analysis of the effect of range condition**

For the 57 participants who had been allocated to different range conditions in different sessions, we used two-way mixed ANOVA to examine the effect of range condition within-subjects (LGR-HLR vs. HGR-LLR) and condition order between-subjects (LGR-HLR first vs. LGR-HLR second) with log(LA_coefficient_) as the dependent measure. The effect of range condition was statistically significant, *F*(1,55) = 9.72, *p* = .003, ${\eta_{p}}^{2}$ = .150, confirming a greater tendency for loss aversion in the HGR-LGR condition (*M* = 0.13, *SD* = 0.34) compared to the LGR-HLR condition (*M* = –0.06, *SD* = 0.31). Neither the effect of condition order nor its interaction with range condition were significant, both *F* < 1, *p* > .65, ${\eta_{p}}^{2}$ < .004. The medians for the LA_coefficient_ illustrate that loss aversion is the norm in the HGR-LLR condition (*Med* = 1.34, CI_95%_[1.12,1.57]) while reverse loss aversion is the norm in the LGR-HLR condition (*Med* = 0.83, CI_95%_[0.74, 0.99]).

**Correlational analysis of stability in loss aversion**

Table S2 reports the correlation for log(LA_coefficient_) across condition pairs, with Pearson correlations reported in the upper right triangle of the matrix and Spearman correlations reported in the lower left triangle. Perhaps unsurprisingly given the relatively small sample size for any given pair of conditions, the strength of these correlations vary quite considerably, and the confidence intervals are wide. However, five of each set of six are positive, and slightly more than half of those represent medium or large effects. Taken together, these correlations provide some evidence for stability in loss aversion, though the strength of correlations are more in line with those for items on an individual difference measure than the test-retest reliabilities for a stable trait dimension.

Table S2. Correlation coefficient (95% CI) for log loss aversion coefficient between condition pairs: Pearson shown top right and Spearman pho shown bottom left.

|  | HA HGR-LLR | LA HGR-LLR | HA LGR-HLR | LA LGR-HLR |
| --- | --- | --- | --- | --- |
| High amount and high gain range with low loss range (HA HGR-LLR) | 1  -- | **.64 (–.30, .94)**  *p* = .006 {n = 17} | **–.33 (–.80, .09)**  *p* = .223 {n =15} | **.47 (–.58, .94)**  *p* = .108 {n = 13} |
| Low amount and high gain range with low loss range (LA HGR-LLR) | **.50 (–.10, .92)**  *p* = .042 | 1  -- | **.03 (–.57, .88)**  *p* = .926 {n = 10} | **.45 (–.12, .76)**  *p* = .056 {n = 19} |
| High amount and low gain range with high loss range (HA LGR-HLR) | **–.50 (–.87, .11)**  *p* = .059 | **.15 (–.63, .85)**  *p* = .688 | 1  -- | **.10 (–.51, .79)**  p = .726 {n = 14} |
| Low amount and high gain range with low loss range (LA LGR-HLR) | **.36 (–.40, .92)**  *p* = .223 | **.24 (–.35, .67)**  *p* = 320 | **.27 (–.35, .69)**  *p* = .350 | 1  -- |

**The effect of Session 1 amount on Session 2 loss aversion by condition**

To further understand the nature and robustness of the effect of Session 1 amount condition on loss aversion in Session 2, we examined each Session 2 condition separately, comparing our loss aversion measures, log(LA_coefficent_) and LA_coefficient_, between the Session 1 amount conditions. Table S3 summarises these analyses. In three of the four conditions, the direction of effect matches that of the overall analysis of this effect (above): with a greater tendency towards loss aversion when participants had made decisions for low amounts of money in their previous session, and (equivalently) a greater tendency towards reverse loss aversion when participants had made decisions for high amounts of money in their previous session. In two of these Session 2 conditions, the effect of Session 1 amount condition is medium in size (${\eta_{p}}^{2}$ > .1), though in no case was the effect statistically significant in any one condition.

Table S3. Effect of amount condition in Session 1 (S1) on degree of loss aversion in Session 2 (S2), examined separately for each condition.

| Condition in Session 2 | Log(LA_coefficient_) in Session 2 | | | | | | |  | Median (95% CI) LA_coefficient_ in S2 | |
| --- | --- | --- | --- | --- | --- | --- | --- | --- | --- | --- |
|  | Mean (SD) | |  | Effect of S1 amount | | | |  |  |  |
|  | S1 low amount | S1 high amount |  | F | df | p | ${\eta_{p}}^{2}$ |  | S1 low amount | S1 high amount |
| High amount and high gain range with low loss range (HA HGR-LLR) | 0.32 (0.46) | –0.05 (0.43) |  | 3.66 | (1,25) | .067 | .128 |  | 1.62 (1.02,2.83) | 1.01  (0.92,1.38) |
| Low amount and high gain range with low loss range (LA HGR-LLR) | 0.12  (0.49) | 0.18  (0.18) |  | 0.14 | (1,21) | .715 | .006 |  | 1.28 (0.80,1.89) | 1.42 (1.06,2.20) |
| High amount and low gain range with high loss range (HA LGR-HLR) | 0.10  (0.55) | –0.07  (0.18) |  | 0.70 | (1,17) | .415 | .039 |  | 1.00  (0.75,1.12) | 0.86  (0.50,1.25) |
| Low amount and high gain range with low loss range (LA LGR-HLR) | –0.02  (0.17) | –0.18  (0.20) |  | 3.07 | (1,17) | .098 | .153 |  | 1.03 (0.72,1.32) | 0.75  (0.53,0.75) |

ANOVA is used to compare amount conditions, rather than *t*-tests, to make it easier to compare the effects with other effects that we report.

**Dominance analysis of the three predictors of loss sensitivity in Session 2**

Table 2 from the manuscript is reproduced below, which shows the three predictors of loss sensitivity that were identified and discussed. Based on the β-coefficients or (equivalently) the R^2^ change values, this analysis suggests that log(LA_coefficient_) in Session 1 (*S1*) might be regarded as the best predictor of log(LA_coefficient_) in Session 2 (*S2*), followed by range condition in *S2* and then amount condition in *S1*. However, this simple reading of Table 2 might not be appropriate because the three predictors are not perfectly orthogonal (see Table S4a). Further analysis was therefore undertaken to better understand the relative contribution of these three variables to the prediction of log(LA_coefficient_) in *S2*.

Following the principles of univariate dominance analysis described by Budescu (1993), we examined the incremental predictive power when each predictor was added (individually) to all possible sub-sets of models that do not contain that predictor. This allowed us to test whether there was a coherent dominance ranking among the predictors, in terms of their contribution to the multivariate prediction of log(LA_coefficient_) in *S2*. Analysis that examines the addition of one predictor to a model already containing the other two predictors was reported in Table 2 of the manuscript (reproduced above). The additional analyses required for univariate dominance analysis of these data are reported in Table S4b. This analysis suggests that a straightforward interpretation of the ranking of *β*-coefficients or R^2^ change values in Table 2 is reasonable, because the following ranking is consistent with each separate piece of analysis: Log(LA_coefficient_) in *S1 >* Range condition in *S2* > Amount condition in *S1*.

*Table 2 (reproduced).* Multiple linear regression with log(LA_coefficient_) in Session 2 as the dependent variable

|  | Regression coefficients | | |  | Unique contribution beyond other predictors | | |
| --- | --- | --- | --- | --- | --- | --- | --- |
| Predictor | Unstandardised *b* [95% CI] | Standardised  $\beta$ | *p*-value |  | R^2^ change | F_change_(1,84) | *p*-value |
| Constant | 0.01  [–0.13,0.16] | -- | .849 |  | -- | -- | -- |
| Log(LA_coefficient_) in Session 1 | 0.51  [0.23,0.78] | 0.35 | < .001 |  | .122 | 13.73 | < .001 |
| Amount condition in Session 1 | –0.19  [–0.35,–0.03] | –0.23 | .020 |  | .050 | 5.63 | .020 |
| Range condition in Session 2 | 0.24  [0.08,0.41] | 0.29 | .003 |  | .081 | 9.05 | .003 |

Amount condition: 0 = low, 1 = high

Range condition: 0 = low gain range with high loss range (LGR-HLR), 1 = high gain range with low loss range (HGR-LLR)

Table S4a. Correlation (*r*) between the variables examined in Table 2, with 95% confidence intervals for r

|  | Log(LA_coefficient_) in Session 2 | Log(LA_coefficient_) in Session 1 | Amount condition in Session 1 | Range condition in Session 2 |
| --- | --- | --- | --- | --- |
| Log(LA_coefficient_) in Session 2 | -- | -- | . -- | -- |
| Log(LA_coefficient_) in Session 1 | .30**  [.05,.51] | -- | -- | -- |
| Amount condition in Session 1 | –.27*  [–0.40,–0.12] | .07  [–.14,.25] | -- | -- |
| Range condition in Session 2 | .29**  [.05,.54] | –.15  [–.34,.06] | –.21*  [–.41,–.01] | -- |

* *p* < .05, ** *p* < .01

Table S4b. Three pairs of hierarchical linear regressions with Log(LA_coefficient_) in Session 2 (S2) as the criterion variable.

| Predictor in subset model at the first step | Predictor added in the second step | R^2^ change | Rank for predictor’s additional contribution to the model |
| --- | --- | --- | --- |
| Log(LA_coefficient_) in S1 | Amount condition in S1 | .083 | Range condition *S2* > Amount condition *S1* |
|  | Range condition in S2 | .114 |  |
| Amount condition in S1 | Log(LA_coefficient_) in S1 | .099 | Log(LA_coefficient_) *S1* > Range condition *S2* |
|  | Range condition in S2 | .057 |  |
| Range condition in S2 | Log(LA_coefficient_) in S1 | .097 | Log(LA_coefficient_) *S1* > Amount condition *S1* |
|  | Amount condition in S1 | .045 |  |

Amount condition: 0 = low, 1 = high

Range condition: 0 = low gain range with high loss range (LGR-HLR), 1 = high gain range with low loss range (HGR-LLR)

**Reference**

Budescu, D. V. (1993). Dominance analysis: A new approach to the problem of relative importance of predictors in multiple regression. *Psychological Bulletin, 114,* 542-551.
